# SGLT2 inhibition slows tumor growth in mice by reversing hyperinsulinemia

**DOI:** 10.1186/s40170-019-0203-1

**Published:** 2019-12-11

**Authors:** Ali R. Nasiri, Marcos R. Rodrigues, Zongyu Li, Brooks P. Leitner, Rachel J. Perry

**Affiliations:** 1 Department of Internal Medicine, School of Medicine, Yale University, PO Box 208020, TAC S269, New Haven, CT 06520 USA; 2 Department of Cellular & Molecular Physiology, School of Medicine Yale University, PO Box 208020, TAC S269, New Haven, CT 06520 USA; 3 Department of Surgery, State University of Ponta Grossa, Ponta Grossa, Brazil

**Keywords:** Tumor metabolism, Obesity, Insulin, Glucose

## Abstract

**Background:**

Obesity confers an increased risk and accelerates the progression of multiple tumor types in rodents and humans, including both breast and colon cancer. Because sustained weight loss is rarely achieved, therapeutic approaches to slow or prevent obesity-associated cancer development have been limited, and mechanistic insights as to the obesity-cancer connection have been lacking.

**Methods:**

E0771 breast tumors and MC38 colon tumors were treated in vivo in mice and in vitro with two mechanistically different insulin-lowering agents, a controlled-release mitochondrial protonophore (CRMP) and sodium-glucose cotransporter-2 (SGLT2) inhibitors, and tumor growth and glucose metabolism were assessed. Groups were compared by ANOVA with Bonferroni’s multiple comparisons test.

**Results:**

Dapagliflozin slows tumor growth in two mouse models (E0771 breast cancer and MC38 colon adenocarcinoma) of obesity-associated cancers in vivo, and a mechanistically different insulin-lowering agent, CRMP, also slowed breast tumor growth through its effect to reverse hyperinsulinemia. In both models and with both agents, tumor glucose uptake and oxidation were not constitutively high, but were hormone-responsive. Restoration of hyperinsulinemia by subcutaneous insulin infusion abrogated the effects of both dapagliflozin and CRMP to slow tumor growth.

**Conclusions:**

Taken together, these data demonstrate that hyperinsulinemia per se promotes both breast and colon cancer progression in obese mice, and highlight SGLT2 inhibitors as a clinically available means of slowing obesity-associated tumor growth due to their glucose- and insulin-lowering effects.

## Background

Approximately 5% of all cancers in men and ~ 10% of all cancers in women are attributable at least in part to obesity [1], with certain tumor types exhibiting an even stronger relationship between excess weight and cancer risk. Among these are breast [2–4] and colon cancer [2, 4, 5], in which obesity promotes tumor appearance, progression, and metastasis in rodent models and in human patients. Hyperinsulinemia, which occurs as a consequence of lipid-induced insulin resistance and may also hasten its progression, is one of many putative links that may explain the association between obesity and cancer: insulin promotes tumor cell division in vitro [6–10], and plasma insulin concentrations are independently correlated with an increased risk and accelerated progression of both breast [11–15] and colon cancer [16–20]. Interfering with insulin signaling may slow tumor growth [21], while activation of the insulin receptor may promote tumor progression [22, 23] in murine models, although there have also been publications demonstrating the absence of an effect of insulin on tumor growth [24]. In humans with type 2 diabetes, exogenous insulin treatment has been linked to an increased risk of developing breast cancer [25–27] and poorer breast cancer outcomes [28–30], although the impact of exogenous insulin on tumor progression is debatable: recent meta-analyses failed to detect an impact of insulin treatment on breast cancer incidence [31–33]. The inconsistent data regarding the impact of exogenous insulin treatment on breast cancer risk may be attributable to the fact that exogenous insulin treatment is designed to restore normal circulating insulin concentrations in those with insufficient β-cell function; thus, it likely does not create hyperinsulinemia in most cases. Even so, the growing body of epidemiologic evidence for an association between insulin and cancer risk and progression begs the question of what explains the potential mechanistic link between obesity, hyperinsulinemia, and cancer.

The ability of insulin to stimulate tumor cell proliferation is likely multifactorial and has been attributed to stimulation of DNA replication and cell cycle progression, potentially by activation of the PI3K/Akt/FOXO1 signaling pathway, increased accumulation of DNA damage, and increases in glucose uptake and/or oxidation. Consistent with the latter mechanism, we have recently shown that insulin’s ability to promote glucose uptake and oxidation in vitro constitutes a metabolic signature of obesity-associated tumor types [6], suggesting that insulin-lowering therapies may be an attractive approach to slow obesity-associated tumor growth, thereby prolonging the window during which curative therapies may be possible.

To that end, we have recently demonstrated that mitochondrial uncoupling with a controlled-release mitochondrial protonophore (CRMP) both prevents and reverses insulin resistance and, as a result, hyperinsulinemia by correcting non-alcoholic fatty liver disease (NAFLD) [34], and that the reversal of hyperinsulinemia slows tumor growth in two mouse models of colon cancer, MC38 tumor-bearing and Apc^Min+/-^ mice [35]. Because uncoupling is not yet an approved pharmacologic strategy to treat NAFLD, it is of great interest to investigate the potential for other agents to reverse obesity-associated insulin resistance, hyperinsulinemia, and accelerated tumor growth. Metformin also slows colon cancer growth in rodents [36–38] in an insulin-dependent manner [35] and may modestly reduce colon cancer incidence in diabetic humans [39, 40]. However, safety concerns exist: metformin can cause undesirable gastrointestinal side effects and is contraindicated in those with limited renal function, as it can cause lactic acidosis. Therefore, there is a strong need to investigate alternative insulin-lowering therapies as a potential adjunct in those with obesity-associated tumors.

SGLT2 inhibitors have recently been investigated as a potential anti-cancer therapy and have been shown to induce apoptosis or inhibit proliferation of breast cancer [41, 42], renal cell carcinoma [43], and hepatocellular carcinoma [44] cells at high concentrations in vitro. In vivo studies in rodents treated with SGLT2 inhibitors have also been promising, if limited: canagliflozin lowered tumor burden in mice with hepatocellular [44–46], lung [47], and renal cell carcinoma [43] in vivo. However, the mechanism by which this occurs—and specifically the possible role of alterations in systemic glucose metabolism and potential ability of changes in plasma insulin concentrations to mediate differences in tumor glucose metabolism—has not been explored in obesity-associated tumor types. Here, we examine the impact of SGLT2 inhibition with dapagliflozin on tumor growth in mouse models of obesity-associated cancers: colon adenocarcinoma (MC38 tumors) and triple-negative breast cancer (E0771 tumors), two commonly used murine cancer models whose driver mutations remain under debate and are likely multifactorial, but which robustly express the insulin receptor [6, 36, 48, 49], and demonstrate that dapagliflozin slows tumor growth in both models. This effect is not due to increases in ketosis or to a direct effect on tumor cell division, but rather is mediated by the reversal of hyperinsulinemia, resulting in reductions in tumor glucose uptake and oxidation. Similarly CRMP slows E0771 tumor growth, similar to its effect to slow colon tumor growth [35], by reversing hyperinsulinemia and insulin-dependent increases in tumor glucose uptake and oxidation, without any direct effect to alter tumor cell metabolism or division. Taken together, these data implicate hyperinsulinemia as a key pathogenic factor in the progression of both breast and colon cancer in two mouse models and identify SGLT2 inhibition as a potential means of slowing obesity-associated cancer progression in vivo.

## Methods

### Cells

E0771 cells were obtained from CH3 Biosystems (Amherst, NY) and cultured in the manufacturer’s recommended media: RPMI 1640 supplemented with 10 mM HEPES, 10% FBS, and antimicrobials (100 U/mL penicillin/100 μg/mL streptomycin/250 μg/mL amphotericin). MC38 cells were obtained from Kerafast (Boston, MA) and cultured in the manufacturer’s recommended media: DMEM containing 25 mM glucose, 2 mM glutamine, 0.1 mM nonessential amino acids, 1 mM sodium pyruvate, 10 mM HEPES, 10% FBS, and antimicrobials (100 U/mL penicillin/100 μg/mL streptomycin/250 μg/mL amphotericin). Neither cell line was authenticated in our laboratory, but all cells were used within 10 passages of obtaining them commercially. Cells were cultured in a 37 °C humidified incubator and split as needed (2–3× weekly). In vitro cell division was measured by incubating 1 × 10^5^ E0771 cells or 5 × 10^4^ MC38 cells in the agents noted for 2 days (or vehicle, the media described above). Forty-eight hours after plating, cells were washed and trypsinized, and live cells were counted by a blinded investigator 48 h later.

### Mice

All animal studies were approved by the Yale University Institutional Animal Care and Use committee. Because E0771 cells are a model of breast cancer that does not express the estrogen or progesterone receptors, and because female mice are protected from obesity-associated insulin resistance [50, 51], male mice were used in all studies. C57bl/6J mice were obtained from Jackson Laboratories at 6–8 weeks of age and after 1 week of acclimation, during which time they were fed regular chow (Envigo 2018S, Huntington, Cambridgeshire, UK), and mice were injected with tumor cells (2 × 10^5^ MC38 cells or 5 × 10^5^ E0771 cells). On the day of injection, treatment with high fat diet (Research Diets D12492, New Brunswick, NJ; 60% kcal from fat, 20% from protein, 20% from carbohydrate) or the same HFD-containing CRMP (7 mg CRMP per gram of diet, with an approximate dose of 12 mg active DNP per kilogram of body weight per day). In the insulin replacement studies, three insulin-containing pellets (LinBit) were implanted subcutaneously on the day of tumor injection. Mice treated with dapagliflozin were given drinking water containing 0.01 mg/mL dapagliflozin (Sigma, St. Louis, MO; approximate dose 2.5 mg/kg per day), and those treated with metformin were given drinking water containing 33 mg/mL metformin (Sigma; approximate dose 200 mg/kg per day). In the β-OHB supplementation study, sodium β-OHB (Sigma) was provided at a concentration of 1 mM (approximate dose: 4 mmol/kg/day).

Tumor size was measured weekly using calipers in duplicate by blinded investigators, with tumors assumed to be spherical for calculation of volume based on the measured diameter. Energetics, food, and water intake were measured using the Columbus Instruments Comprehensive Lab Animal Monitoring System (CLAMS; Columbus, OH) during the first week after tumor implantation. Body composition (lean and fat mass) were examined by nuclear magnetic resonance spectroscopy (Bruker minispec; Billerica, MA). Mice were sacrificed 3–4 weeks after tumor implantation, or when tumor size exceeded 2000 mm^3^. One week prior to tracer studies, mice underwent surgery to place catheters in the right jugular vein, which were then advanced into the right atrium. After recovery of their pre-surgical body weight and following a 5-h fast, mice underwent tracer studies as described below. Mice were sacrificed at the end of the tracer studies using IV pentobarbital. Tumor, liver, and skeletal muscle (gastrocnemius) were harvested in N_2_-cooled freeze clamps and stored at – 80 °C to await further analysis. In the oral glucose tolerance tests (OGTT), mice were gavaged with 1 g/kg dextrose, and a blood sample was taken from the tail vein 0 min (no gavage), 15, 30, 60, or 120 min afterward. The mouse was sacrificed with isoflurane anesthesia immediately after obtaining the blood sample, and tumors were isolated.

### Assessment of tumor glucose uptake and oxidation

Tumor glucose uptake and *V*_PDH_/*V*_CS_ were measured ex vivo after a 6-h fast following a steady-state infusion of [U-^13^C_6_] glucose (1 mg/kg/min following a 5-min 3X prime) and a bolus injection of [1-^14^C] 2-deoxy-d-glucose [35]. Briefly, *V*_PDH_/*V*_CS_ was measured in vivo and in vitro as the ratio of [4,5-^13^C_2_] glutamate/[^13^C_3_] alanine, with glutamate enrichment measured by LC-MS/MS and alanine enrichment by GC/MS. To measure *V*_PDH_/*V*_CS_ in vitro*,* we incubated 1 × 10^5^ MC38 cells or 2 × 10^5^ E0771 cells in a 6-well plate for 120 min in the manufacturer’s recommended media, described above, modified to supply physiological concentrations of glucose (5 mM [U-^13^C_6_] glucose), and physiological fatty acids (1 mM potassium palmitate). After 120 min, 1 mL 50% methanol was added, and cells were scraped, transferred to a 1.5 mL Eppendorf tube, centrifuged, and processed to measure *V*_PDH_/*V*_CS_ as described above. Of note, we have previously demonstrated that physiologic concentrations of glutamine do not significantly confound the measurement of this ratio [6]. Where indicated, insulin, dapagliflozin, canagliflozin (Sigma), DNP (Sigma), or β-OHB (Sigma) were added at the concentrations specified. To measure glucose uptake in vitro, [^14^C] 2-deoxyglucose (0.1 μCi) was added to cells (2 × 10^5^ E0771 cells or 1 × 10^5^ MC38 cells) in media described above. Thirty minutes later, cells were washed three times in warmed PBS, and scraped, collected in a scintillation vial, and [^14^C] specific activity determined using a scintillation counter. The rate of glucose uptake was calculated assuming a constant rate of glucose uptake over the 30-min incubation period.

### Biochemical analysis

Plasma and urine glucose concentrations were measured using the YSI Glucose Analyzer. Plasma insulin was measured by RIA by the Yale Diabetes Research Center, or (OGTT samples only) by ELISA (Mercodia, Uppsala, Sweden), and plasma β-OHB by GC/MS, using the sample preparation method we have described [52] after spiking plasma samples with a 1-mM [^13^C_4_] β-OHB standard. Tumor DNP concentrations were assessed by LC-MS/MS [53]. Tumor Akt pSer473 phosphorylation and total Akt expression, pS70 S6K pThr389 phosphorylation, and total S70 S6K expression were assessed by western blot using antibodies from Cell Signaling (catalog numbers 9271, 2920, 9206, and 9202, respectively).

### Statistical analysis

GraphPad Prism 7.0 was used for statistical analysis. Two groups were compared by the two-tailed unpaired Student’s *t* test, and three or more groups by ANOVA with Bonferroni’s multiple comparisons test, after verifying that the data met the assumptions of the statistical test employed. Data are presented as the mean ± S.E.M.

## Results

### Dapagliflozin slows E0771 tumor growth in obese mice in an insulin-dependent manner

**Fig 1:**
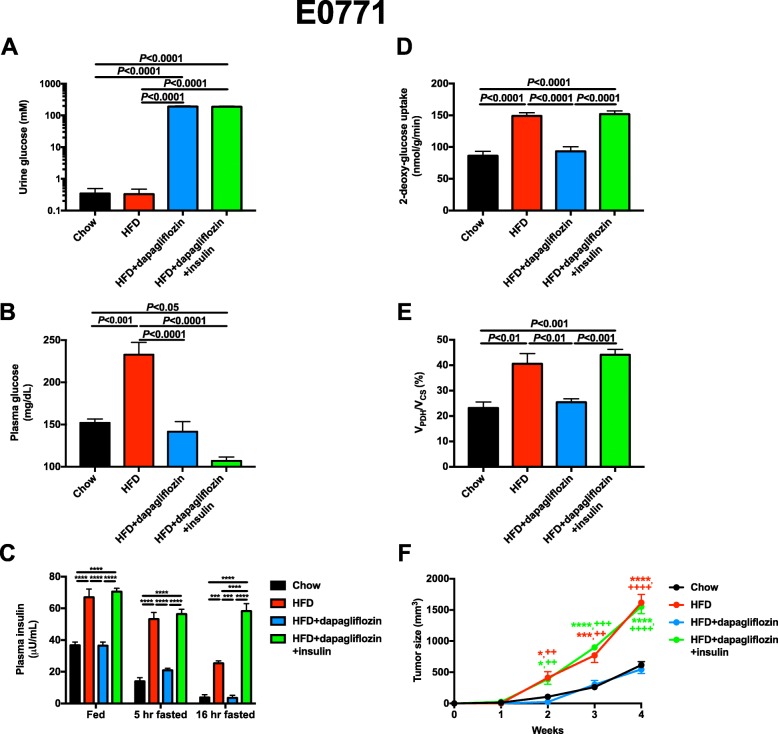
Dapagliflozin slows E0771 breast tumor growth in an insulin-dependent manner. **a**, **b** Urine and plasma glucose concentrations. Unless otherwise designated, all measurements were performed in 5-h fasted mice. **c** Plasma insulin. **d**, **e** Tumor 2-deoxyglucose uptake and *V*_PDH_/*V*_CS_. **f** Tumor size. *P < 0.05, ***P < 0.001, ****P < 0.0001 vs. chow, ++*P* < 0.01, ++++*P* < 0.0001 vs. HFD + dapagliflozin, with the color of the symbols indicating the group compared to the group designated by the symbols. In all panels, data are the mean ± S.E.M. of *n* = 5 per group. Groups were compared by ANOVA with Bonferroni’s multiple comparisons test

To examine the potential utility of dapagliflozin as an anti-tumor agent in vivo, we treated obese mice with dapagliflozin in drinking water beginning on the day of E0771 tumor implantation. Not surprisingly, dapagliflozin caused glycosuria, but did not affect energy expenditure or caloric intake, measured during the first week of treatment before the groups of mice diverged in body weight (Fig. 1a, Additional file 1: Figure S1A-J). As expected, water intake increased in the dapagliflozin-treated group as a compensatory mechanism to avoid dehydration, and a small (1%), physiologically insignificant increase in respiratory exchange ratio was also observed. However, 3 weeks later, sustained glucose wasting in urine was associated with reductions in body weight and fat mass in high-fat fed mice (Additional file 1: Figure S1K-L). SGLT2 inhibition lowered plasma glucose concentrations in 5-h fasted mice by 80 mg/dL and reduced plasma insulin concentrations in fed, 5-h fasted, and 16-h fasted mice (Fig. 1b, c), in contrast to metformin, which lowered plasma insulin only after a prolonged fast (Additional file 1: Figure S1M). To examine the impact of the reduction in plasma insulin on tumor growth and metabolism, we infused insulin subcutaneously to match plasma insulin concentrations in 5-h fasted dapagliflozin-treated mice to those measured in untreated HFD controls. E0771 tumor glucose metabolism was insulin-responsive: glucose uptake and oxidation were increased in tumors of HFD fed, hyperinsulinemic mice but normalized with dapagliflozin treatment; however, restoring hyperinsulinemia via subcutaneous insulin infusion increased tumor glucose uptake and oxidation to rates observed in HFD control mice. Hyperinsulinemia had a profound effect on tumor growth rates: 4 weeks after tumor implantation, E0771 tumors were 1000 mm^3^ larger in HFD mice than lean controls. However, dapagliflozin treatment reduced rates of tumor growth such that tumor growth in dapagliflozin-treated mice mimicked that of chow fed animals. This effect was insulin-mediated: restoring hyperinsulinemia increased tumor growth rates in dapagliflozin-treated mice to those measured in obese HFD mice.

**Fig. 2:**
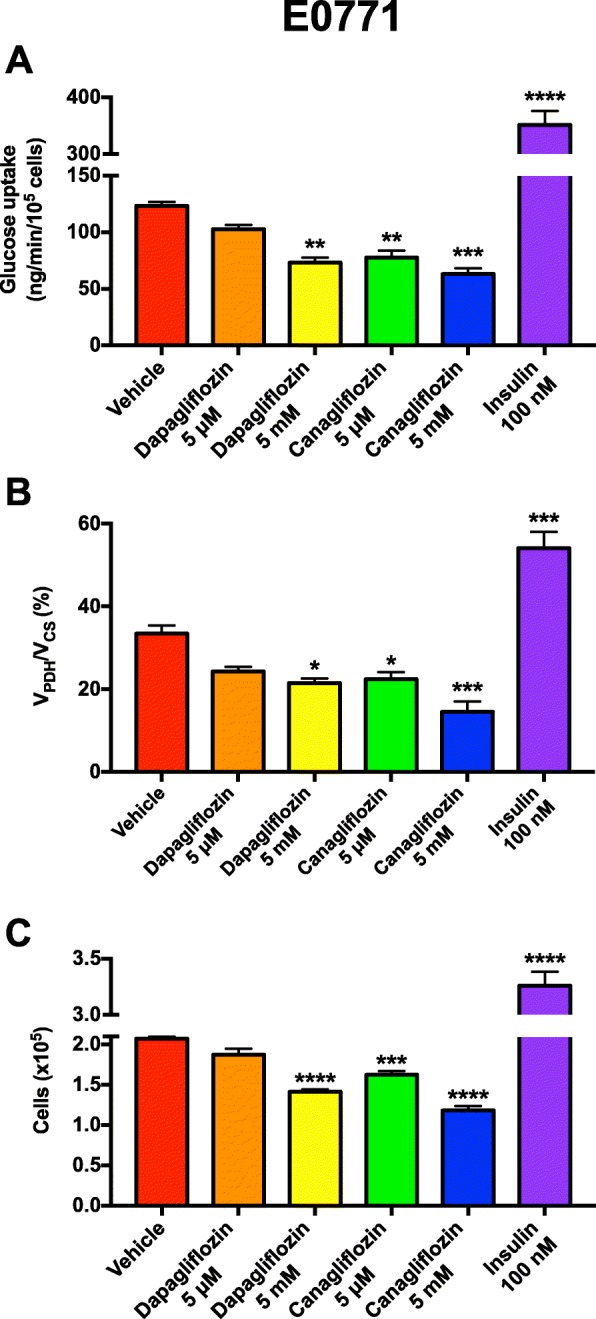
Impact of SGLT2 inhibitors and insulin on E0771 cell metabolism and division in vitro. **a** Glucose uptake. **b** *V*_PDH_/*V*_CS_. **c** Cell division. In all cell studies, data are the mean ± S.E.M. of *n* = 4 replicates. Data were compared by ANOVA with Bonferroni’s multiple comparisons test, in which each group was compared to the mean of the vehicle-treated cells

Next, we aimed to examine whether the ability of dapagliflozin to slow E0771 tumor growth was a cell-autonomous effect. The maximum daily dose of dapagliflozin is 10 mg per day in humans; this dose results in a peak dapagliflozin concentration of less than 0.4 μM [54]. Ten-fold higher dapagliflozin concentrations had no impact on E0771 tumor glucose uptake or oxidation, nor did this dose of dapagliflozin alter cell division in vitro (Fig. 2a–c); however, 10,000-fold higher, suprapharmacologic dapagliflozin concentrations did reduce glucose uptake and oxidation, associated with slower E0771 cell division in vitro. In contrast, canagliflozin showed a dose-dependent effect to reduce tumor glucose uptake and oxidation and to suppress tumor cell division at pharmacologically relevant doses [55] (Fig. 2a–c). These data indicate that SGLT2 inhibitors may have some cell-autonomous effect to slow tumor growth, likely through direct suppression of tumor glucose metabolism. However, the lack of an effect of dapagliflozin at pharmacologically relevant concentrations suggests that most if not all of the impact of this agent occurs through alterations in systemic metabolism. In contrast, insulin—at doses that are supraphysiologic but commonly used in in vitro studies in the literature—promoted both glucose uptake and oxidation in E0771 tumors, accelerating tumor cell division (Fig. 2a–c).

### CRMP slows E0771 tumor growth in obese mice in an insulin-dependent manner

**Fig. 3:**
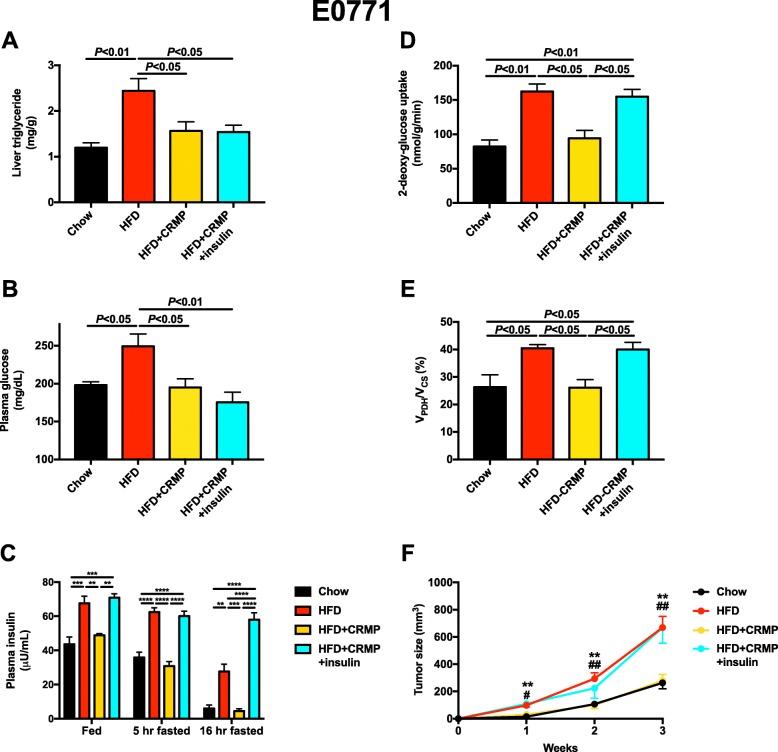
A controlled-release mitochondrial protonophore slows E0771 breast tumor growth in an insulin-dependent manner. **a** Liver triglyceride content. In all panels, unless otherwise specified, all mice were fasted for 5 h before they were studied. **b**, **c** Plasma glucose and insulin concentrations. **d** Tumor 2-deoxyglucose uptake and *V*_PDH_/*V*_CS_. **f** Tumor size. **P < 0.01 vs. chow, #*P* < 0.05, ##*P* < 0.01 vs. HFD + CRMP, with the color of the symbols indicating the group compared to the group designated by the symbols. In all panels, data are the mean ± S.E.M. of *n* = 5–6 per group. Groups were compared by ANOVA with Bonferroni’s multiple comparisons test

After observing the ability of dapagliflozin to slow E0771 breast tumor growth by reversing hyperinsulinemia, we asked whether similar effects would be seen as a result of reducing circulating insulin concentrations with an agent that works through a divergent mechanism. To that end, we treated obese, HFD fed, E0771 tumor-bearing mice with CRMP. Consistent with previous data [34, 56] and with an uncoupling effect confined to the liver, CRMP treatment did not alter body weight or fat mass, whole-body energy expenditure, food or water intake, or respiratory exchange ratio in obese mice (Additional file 1: Figure S2A-J). However, mitochondrial uncoupling lowered liver, plasma, and skeletal muscle triglyceride content (Fig. 3a, Additional file 1: Figure S2K-L). This reduction in ectopic lipid content resulted in lower 5-h fasted plasma glucose concentrations, and lower plasma insulin concentrations under both fed and fasted conditions (Fig. 3b, c). Tumor glucose uptake and oxidation were both modulated by circulating insulin concentrations and normalized by insulin sensitization: high fat feeding increased, and CRMP decreased, both parameters. However, restoring hyperinsulinemia by chronic subcutaneous insulin infusion increased tumor glucose uptake and oxidation to levels measured in untreated HFD tumor-bearing mice, confirming that tumor glucose uptake and oxidation are dynamic and insulin-responsive (Fig. 3d, e). These insulin-mediated alterations in tumor glucose metabolism translated to differences in tumor size: high fat feeding accelerated E0771 tumor growth, whereas the reversal of insulin resistance and hyperinsulinemia with CRMP reversed this effect through an insulin-mediated mechanism. However, infusion of insulin via subcutaneous pellet to match plasma insulin concentrations in CRMP-treated mice to those of HFD control animals completely abrogated the effect of CRMP to slow tumor growth (Fig. 3f).

**Fig. 4:**
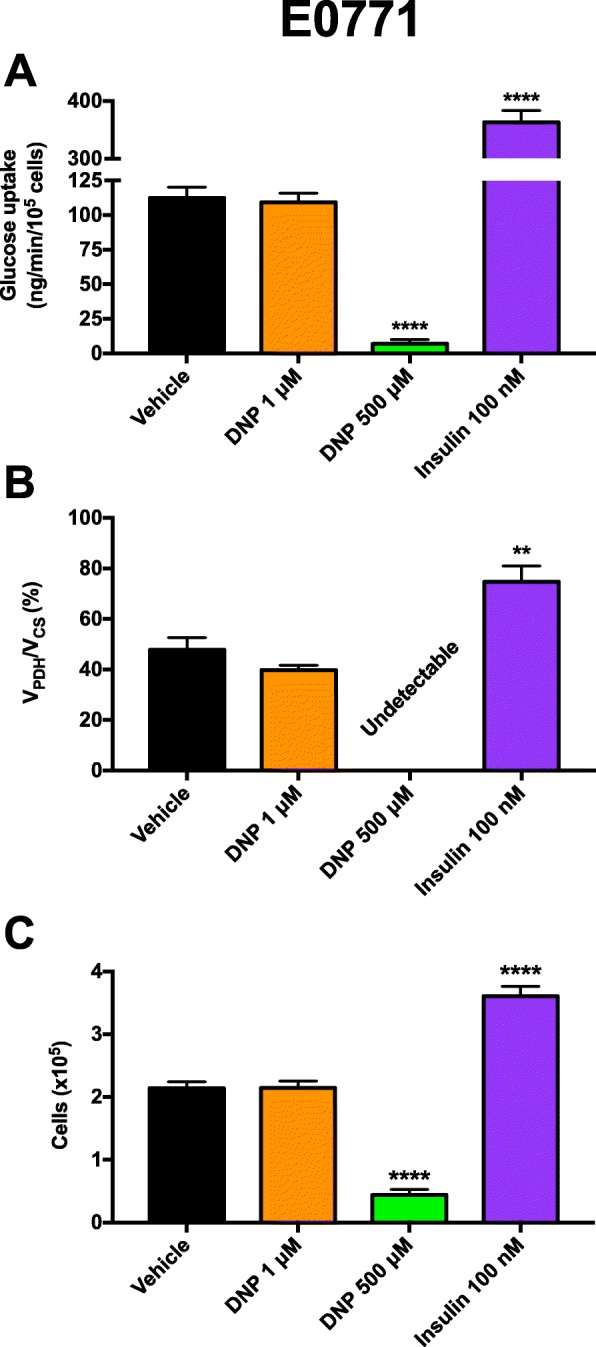
Impact of DNP and insulin on E0771 cell metabolism and division in vitro. **a** Glucose uptake. **b** *V*_PDH_/*V*_CS_. In all samples, this ratio was below the limit of detection (0.5%) in 500 μM DNP-treated cells. **c** Cell division. In all cell studies, data are the mean ± S.E.M. of *n* = 4 replicates. Data were compared by ANOVA with Bonferroni’s multiple comparisons test, in which each group was compared to the mean of the vehicle-treated cells

Next, we aimed to understand whether DNP exerted a direct effect on tumor growth or metabolism independent of insulin. Although tumor DNP concentrations in six CRMP-treated mouse tumors were negligible (0.015 ± 0.003 nmol/g, approximately equivalent to 0.015 μM), we confirmed that DNP did not affect tumor glucose metabolism or growth directly by measuring the rate of glucose uptake, *V*_PDH_/*V*_CS_, and cell division in vitro in MC38 cells and found each parameter to be unaltered after incubation in 1 μM DNP but increased with high concentrations of insulin (Fig. 4a–c). However, higher, markedly supraphamacologic DNP concentrations were toxic to the cells, reducing tumor glucose uptake, oxidation, and cell number.

### Dapagliflozin slows MC38 tumor growth in obese mice in an insulin-dependent manner

**Fig. 5:**
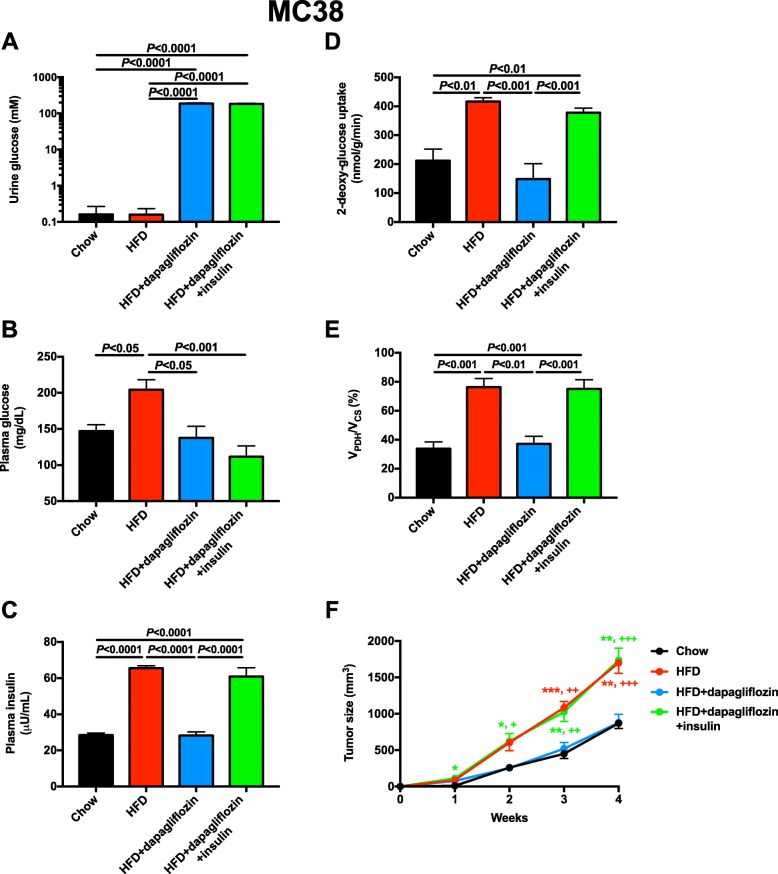
Dapagliflozin slows MC38 colon adenocarcinoma tumor growth in an insulin-dependent manner. **a**, **b** Urine and plasma glucose concentrations. In all panels, mice were fasted for 5 h before studies were performed. **c** Plasma insulin concentrations. **d**, **e** Tumor glucose uptake and *V*_PDH_/*V*_CS_. **f** Tumor size. *P < 0.05, **P < 0.01, ***P < 0.001 vs. chow; +*P* < 0.05, ++*P* < 0.01, +++*P* < 0.001 vs. HFD + dapagliflozin. The color of the symbols indicates the group compared to the group designated by the symbols. In all panels, data are the mean ± S.E.M. of *n* = 5–6 per group, with groups compared by ANOVA with Bonferroni’s multiple comparisons test

Having demonstrated that dapagliflozin impedes E0771 tumor growth by reversing systemic hyperinsulinemia, we next asked whether these results would translate to a second obesity-associated mouse tumor model: MC38 colon adenocarcinoma. Dapagliflozin caused profound glucosuria and increased water drinking before divergence in body weight, but did not affect food intake, energy expenditure, or the respiratory exchange ratio (Additional file 1: Figure S3A-J). However, after 4 weeks of treatment, dapagliflozin treatment resulted in lower body weight, an effect partially abrogated by insulin replacement (Additional file 1: Figure S3K-L). Chronic dapagliflozin treatment lowered plasma glucose and insulin concentrations and reduced both tumor glucose uptake and *V*_PDH_/*V*_CS_ in an insulin-dependent manner, whereas obesity increased tumor glucose metabolism and SGLT2 inhibition normalized it, the obesity- and hyperinsulinemia-associated increases in glucose uptake and oxidation were restored by chronic subcutaneous insulin infusion (Fig. 5b–e). Similar to its effect in E0771 breast cancer, dapagliflozin slowed MC38 tumor growth: 4 weeks after tumor implantation, tumor size was reduced by 50% in dapagliflozin-treated obese mice; however, the effect of dapagliflozin to slow tumor growth was reversed by restoring hyperinsulinemia (Fig. 5f).

**Fig. 6:**
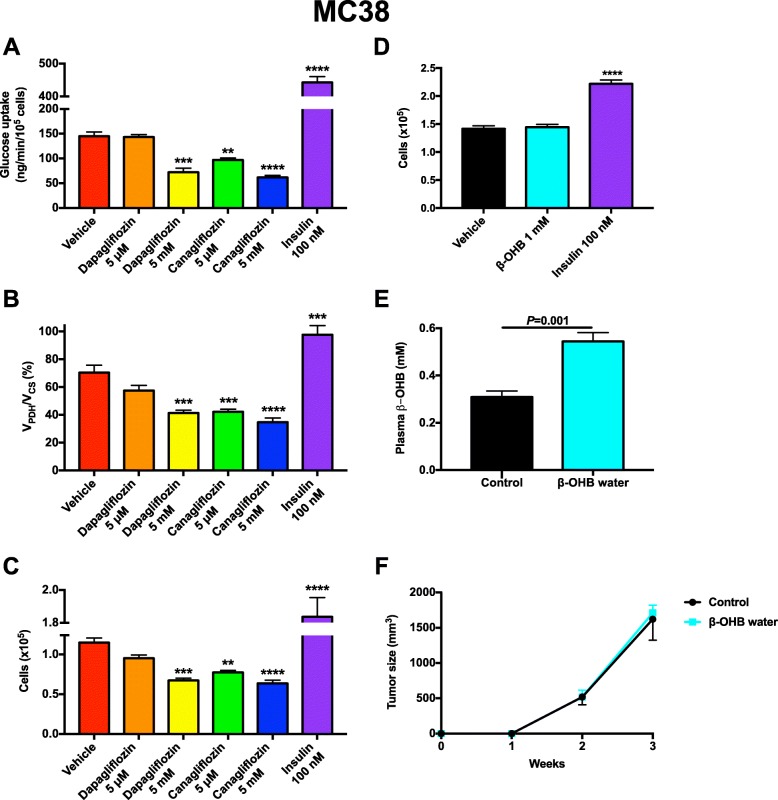
Neither SGLT2 inhibitors nor β-OHB directly alter MC38 tumor cell division. **a** Impact of SGLT2 inhibitors and insulin on MC38 cell glucose uptake in vitro. In panels **a**–**d**, *n* = 4 in vitro replicates, with groups compared by ANOVA with Bonferroni’s multiple comparisons test, in which each group was compared to the vehicle-treated cells (**P < 0.01, ***P < 0.001, ****P < 0.0001). **b** *V*_PDH_/*V*_CS_. **c** Cell division. **d** Impact of β-OHB and insulin on cell division. **e** Plasma β-OHB concentrations after 4 weeks of β-OHB supplementation in drinking water, which was removed 2 h before plasma was obtained. **f** Tumor size. In panels **e** and **f**, *n* = 8 per group, with groups compared by the two-tailed unpaired Student’s *t* test. All data are presented as the mean ± S.E.M

Next, we explored whether SGLT2 inhibitors exert a direct effect to alter glucose metabolism or cell division in MC38 cells at pharmacologically relevant concentrations. While dapagliflozin did not alter glucose uptake, *V*_PDH_/*V*_CS_, or cell division at concentrations in the range of those measured in patients treated with the maximum daily dose of the drug (0.5 μM), but reduced all three parameters at a suprapharmacologic concentration (5 mM), canagliflozin reduced both glucose uptake and oxidation at pharmacologically relevant concentrations (Fig. 6a–c). Because SGLT2 inhibitors can cause ketoacidosis [57], albeit typically not in the well-hydrated state [58], and a ketogenic diet may slow tumor growth, at least in animals [59], we then asked whether ketones themselves may alter tumor growth. In contrast to insulin, which promoted MC38 cell division in vitro at supraphysiologic concentrations, incubation in physiologic (1 mM) concentrations of β-hydroxybutyrate (β-OHB) had no impact on cell division (Fig. 6d). Finally, we examined the potential impact of ketones themselves to alter MC38 tumor growth in vivo and found that chronic (4 weeks) ketone supplementation in drinking water had no impact on plasma glucose or insulin concentrations or on tumor size, despite doubling plasma β-OHB concentrations (Fig. 6e, f, Additional file 1: Figure S3M-N).

### Tumor insulin signaling changes dynamically after a meal

**Fig. 7:**
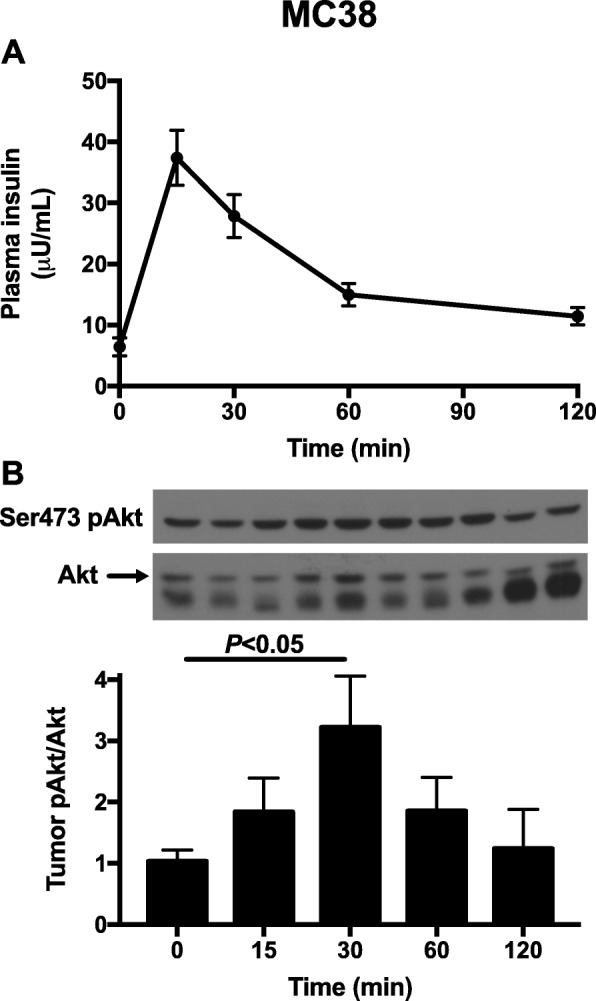
Insulin signaling is dynamically activated under postprandial conditions in MC38 tumors. **a** Plasma insulin concentrations during an oral glucose tolerance test. In both panels, *n* = 4–10 per time point. **b** Tumor Akt pSer473 phosphorylation during the glucose tolerance test. All groups were compared to time zero by ANOVA with Bonferroni’s multiple comparisons test. Data are the mean ± S.E.M

The fact that insulin promotes tumor growth in vivo, associated with increased tumor glucose uptake and oxidation, and that two agents that lower both fasting and postprandial insulin concentrations strikingly slow both E0771 and MC38 tumor growth, suggest that tumor insulin signaling may be dynamically regulated. To test this possibility, we performed an oral glucose tolerance test in MC38 tumor-bearing mice and observed a transient postprandial increase in phosphorylation of Akt in tumor (pSer473), which was slightly delayed as compared to plasma insulin concentrations, and an increase in tumor pThr389 p70 S6K (Fig. 7a, b, Additional file 1: Figure S5A-B), demonstrating that tumor insulin signaling indeed is acutely altered in response to normal physiological changes in insulin concentrations.

## Discussion

Obesity increases the risk of certain tumor types, and intentional weight loss may mitigate cancer risk [60, 61]; however, because weight loss is difficult to achieve and even more difficult to maintain, and may not be advisable in certain patients with cancer, alternative approaches—and inquiry into the mechanisms by which obesity-associated cancer risk may be reduced—are urgently warranted. Metformin has been shown to slow both breast [62] and colon cancer [63] in multiple preclinical models, but results in humans are mixed and clear evidence for a large anti-tumor effect of metformin are lacking. Metformin’s underwhelming efficacy may be due in part to its mechanism: the drug inhibits gluconeogenesis but does not exert an insulin-sensitizing effect; therefore, it lowers fasting plasma glucose and insulin concentrations but has a much smaller effect on these parameters under postprandial conditions (Additional file 1: Figure S1M). In the current study, we demonstrate that tumor insulin signaling is dynamically regulated under postprandial conditions (Fig. 7). These data suggest that agents that lower both fasting and postprandial insulin concentrations, whether by urinary glucose wasting (Fig. 1a,c, and Fig. 5a,c) or by insulin sensitization (Fig. 3c) would be more effective because they would have a more profound impact on total insulin area under the curve throughout the day.

For this reason, in the current study, we investigated the impact of two agents that lower plasma insulin concentrations under both fed and fasted conditions, CRMP and dapagliflozin, in two mouse models of obesity-associated cancer. Previous studies have reported conflicting results regarding the interaction between SGLT2 inhibitors and cancer risk: a slightly—not statistically significantly—elevated risk of bladder cancer in men and of breast cancer in women was initially reported; however, this has since been attributed to detection bias, as both animal studies using higher doses and better-powered human studies have not detected an increased risk of cancer in those treated with SGLT2 inhibitors [64]. To the contrary, recent in vivo studies have suggested that SGLT2 inhibition may be an effective means of attenuating hepatocellular [44–46], renal cell [43], and lung cancer [47] growth by an unclear mechanism.

Based on the insulin-dependent impact of a mitochondrial uncoupler to slow obesity-associated colon cancer growth in mouse models [35], we hypothesized that the SGLT2 inhibitor dapagliflozin, which lowers both fasting and postprandial glucose and insulin concentrations, may slow obesity-associated breast and colon tumor growth through an insulin-dependent mechanism. Consistent with this hypothesis, dapagliflozin slowed tumor growth in mouse models of obesity-associated breast and colon cancer; however, the ability of dapagliflozin to slow tumor growth was completely abrogated by subcutaneous insulin infusion to increase plasma insulin concentrations to those measured in HFD controls. While suprapharmacologic concentrations of two SGLT2 inhibitors did reduce E0771 and MC38 tumor cell division in a cell-autonomous manner, this effect was correlated with reductions in glucose uptake and oxidation in vitro—again demonstrating the key role for dynamic regulation of tumor glucose uptake—and is likely of minimal relevance in vivo; otherwise, dapagliflozin would be expected to slow tumor growth independent of insulin replacement. Importantly, an insulin-dependent or insulin-independent effect on tumor glycolysis and/or lactate metabolism cannot be ruled out in these studies: it is possible that insulin-dependent changes in tumor cell glucose oxidation combine with alterations in glycolytic metabolism to mediate the effects observed on tumor growth in vivo.

Having demonstrated that dapagliflozin slows tumor growth in two mouse models of obesity-associated cancer and that reversal of hyperinsulinemia is necessary for the in vivo tumor-suppressive effect of this SGLT2 inhibitor, we then examined a second insulin-lowering agent, CRMP, which works by an entirely independent mechanism: insulin sensitization [34], rather than calorie loss through glycosuria. CRMP reversed NAFLD and normalized muscle lipid content, likely through reductions in hepatic lipid export, as we have previously demonstrated [34]. Like dapagliflozin, incubation in DNP, the active agent in CRMP, had no impact on tumor cell division in vitro. Combined with in vivo data demonstrating that increasing plasma insulin concentrations in CRMP-treated mice to match those measured in HFD controls renders CRMP unable to slow tumor progression, these data demonstrate that the ability of this insulin sensitizer to slow E0771 breast tumor growth depends on its effect to reverse hyperinsulinemia. In addition, changes in body weight and body fat were dissociated from the effect of CRMP on tumor growth: CRMP treatment was not associated with any difference in either parameter, despite its striking effect to slow tumor growth.

## Conclusions

In summary, these data demonstrate that strategies to mitigate hyperinsulinemia—preferably both fasting and postprandial hyperinsulinemia, as can be accomplished using SGLT2 inhibitors, which are already in the clinic, or insulin sensitizers under development—may be therapeutic targets worthy of further exploration to attenuate breast and/or colon cancer risk and progression. These results are consistent with clinical data indicating that exercise and weight loss, which are classic insulin sensitizing interventions, attenuate the risk and slow the progression of breast cancer [60, 65, 66] and may also slow the progression of colon cancer [67–69]. However, because weight loss after cancer diagnosis is associated with poorer outcomes, interventions that cause little to no weight loss but reduce insulin concentrations out of proportion to body weight change may be attractive targets. Taken together, these data demonstrate that hyperinsulinemia per se promotes breast cancer progression in obese mice, and recommend clinical studies investigating the potential utility of insulin-lowering agents including SGLT2 inhibitors and insulin-sensitizing mitochondrial protonophores currently under development as a means of slowing obesity-associated breast tumor growth.

## Supplementary information

**Additional file 1: Figure S1.** Dapagliflozin slows E0771 breast tumor growth in an insulin-dependent manner. **Figure S2.** A controlled-release mitochondrial protonophore slows E0771 breast tumor growth in an insulin-dependent manner. **Figure S3.** Dapagliflozin slows MC38 colon tumor growth in an insulin-dependent manner. **Figure S4.** Ketone supplementation in drinking water does not independently alter MC38 colon tumor growth. **Figure S5.** Insulin signaling is dynamically activated under postprandial conditions in MC38 tumors.

## Data Availability

The data generated in this study are available from the corresponding author upon reasonable request.

## Abbreviations

CRMP: Controlled-release mitochondrial protonophore
NAFLD: Non-alcoholic fatty liver disease
SGLT2: Sodium-glucose cotransporter-2
