# Supplementary material for: SGLT2 inhibition slows tumor growth in mice by reversing hyperinsulinemia
Source: Cancer Metab. 2019 Dec 11;7:10. doi: 10.1186/s40170-019-0203-1 (PMC6907191; doi:10.1186/s40170-019-0203-1)
Supplement: Supplementary file 1 — Additional file 1: Figure S1. Dapagliflozin slows E0771 breast tumor growth in an insulin-dependent manner. Figure S2. A controlled-release mitochondrial protonophore slows E0771 breast tumor growth in an insulin-dependent manner. Figure S3. Dapagliflozin slows MC38 colon tumor growth in an insulin-dependent manner. Figure S4. Ketone supplementation in drinking water does not independently alter MC38 colon tumor growth. Figure S5. Insulin signaling is dynamically activated under postprandial conditions in MC38 tumors. [file 40170_2019_203_MOESM1_ESM.pdf]

Figure S1

E0771

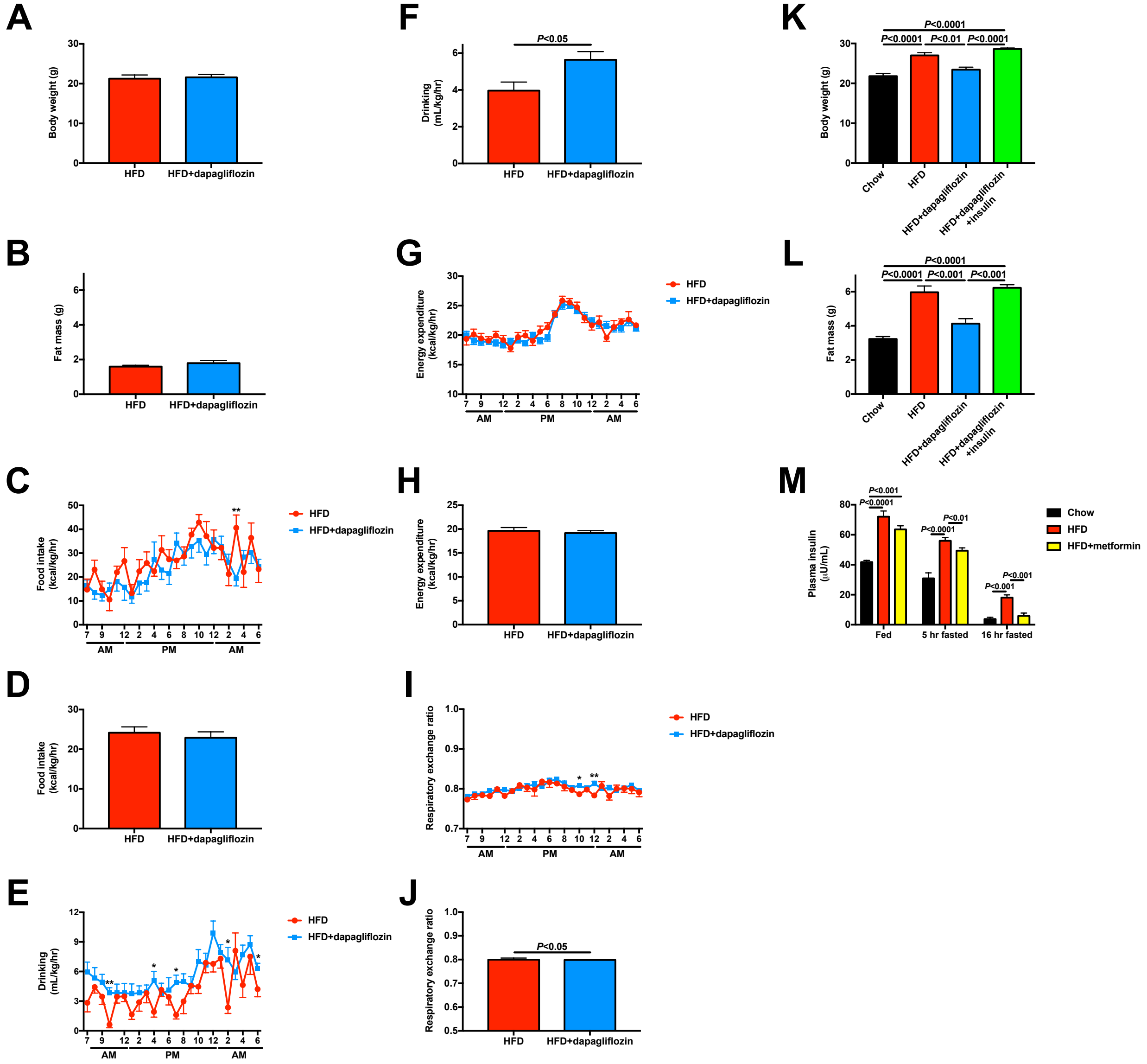

Figure S1. Dapagliflozin slows E0771 breast tumor growth in an insulin-dependent manner. (A)-(B) Body weight and fat mass one week after tumor implantation. (C) Food intake timecourse. In panels (A)-(J), **P*<0.05, ***P*<0.01, ****P*<0.001, *****P*<0.0001 by the 2-tailed unpaired Student's t-test. (D) Daily food intake. (E)-(F) Water drinking timecourse and daily water drinking. (G)-(H) Energy expenditure timecourse and daily energy expenditure. (I)-(J) Respiratory exchange ratio timecourse and daily respiratory exchange ratio. (K)-(L) Body weight and fat mass four weeks after tumor implantation and initiation of HFD±dapagliflozin. Groups in panels (K)-(M) were compared by ANOVA with Bonferroni's multiple comparisons test. (M) Plasma insulin concentrations in tumor-bearing mice treated chronically with metformin in drinking water. All data are the mean±S.E.M. of n=5-12 per group.

Figure S2

E0771

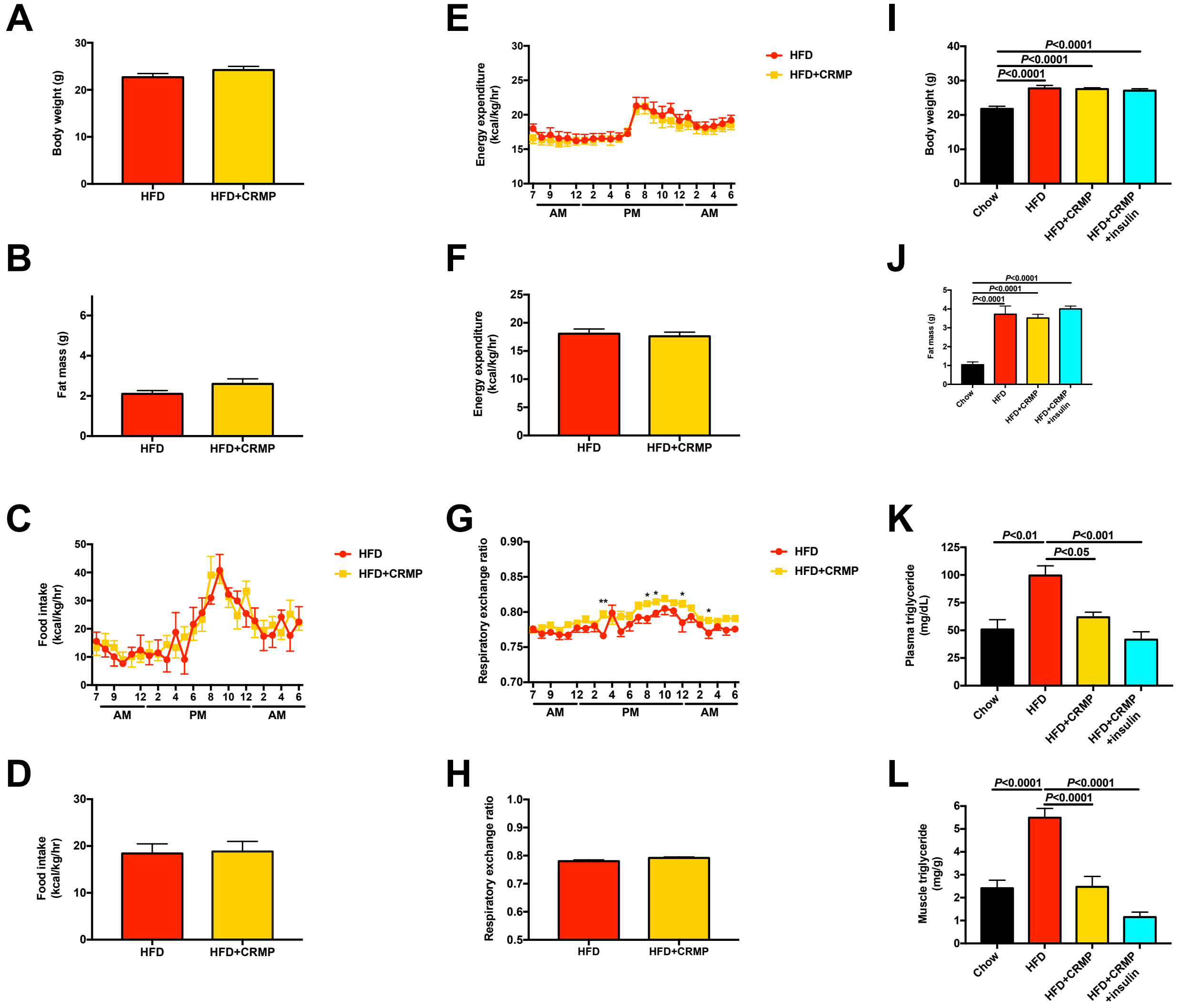

Figure S2. A controlled-release mitochondrial protonophore slows E0771 breast tumor growth in an insulin-dependent manner. (A)-(B) Body weight and fat mass one week after tumor implantation. (C) Food intake timecourse. In panels (A)-(H), **P*<0.05, ***P*<0.01 by the 2-tailed unpaired Student's t-test. (D) Daily food intake. (E)-(F) Energy expenditure timecourse and daily energy expenditure. (G)-(H) Respiratory exchange ratio timecourse and daily respiratory exchange ratio. (I)-(J) Body weight and fat mass four weeks after tumor implantation and initiation of HFD±CRMP±insulin. Groups in panels (I)-(L) were compared by ANOVA with Bonferroni's multiple comparisons test. (K)-(L) Plasma and gastrocnemius triglyceride concentrations. All data are the mean±S.E.M. of n=5-11 per group.

Figure S3 MC38

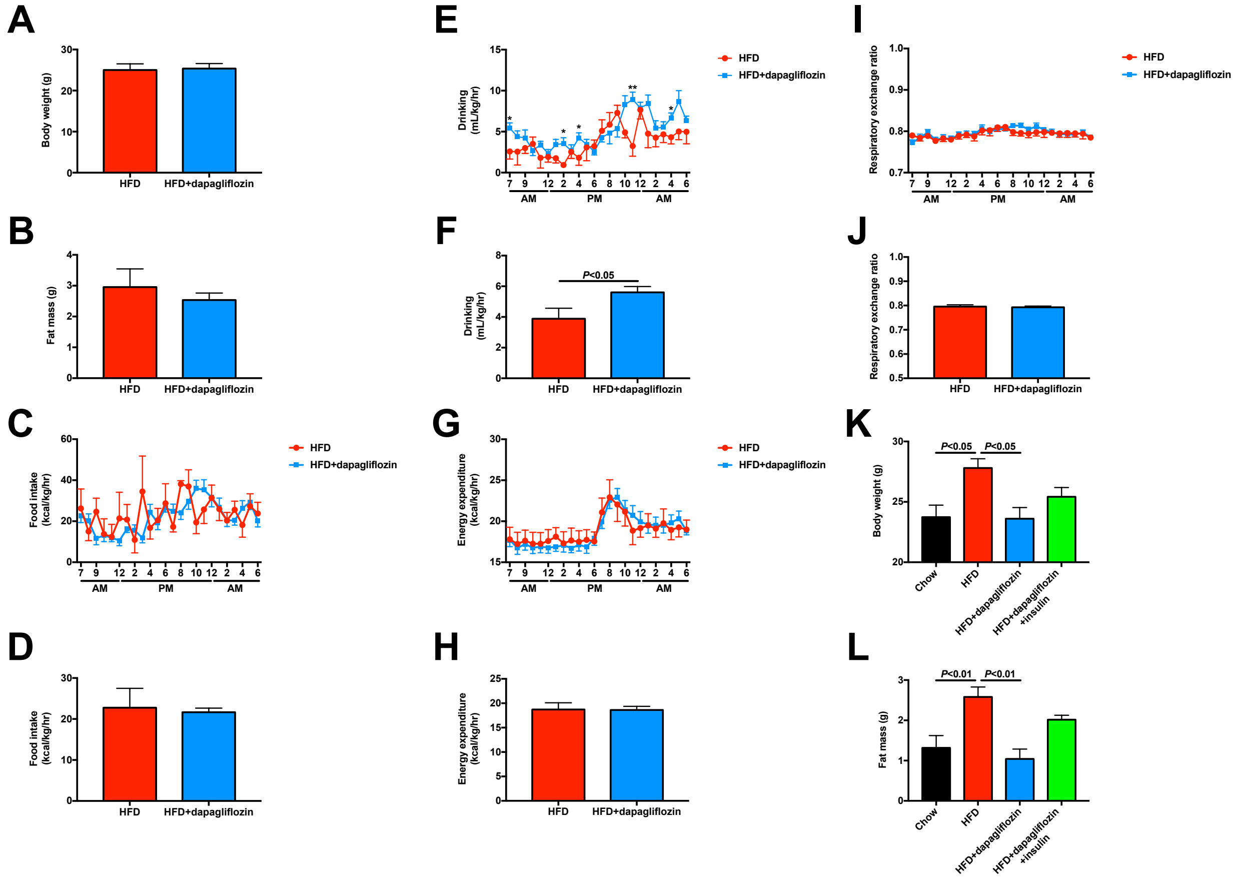

Figure S3. Dapagliflozin slows MC38 colon tumor growth in an insulin-dependent manner. (A)-(B) Body weight and fat mass one week after tumor implantation. (C) Food intake timecourse. In panels (A)-(J), **P*<0.05, ***P*<0.01, ****P*<0.001, *****P*<0.0001 by the 2-tailed unpaired Student's t-test. (D) Daily food intake. (E)-(F) Water drinking timecourse and daily water drinking. (G)-(H) Energy expenditure timecourse and daily energy expenditure. (I)-(J) Respiratory exchange ratio timecourse and daily respiratory exchange ratio. (K)-(L) Body weight and fat mass four weeks after tumor implantation and initiation of HFD±dapagliflozin. Groups in panels (K)-(L) were compared by ANOVA with Bonferroni's multiple comparisons test. All data are the mean±S.E.M. of n=5-12 per group.

Figure S4

MC38

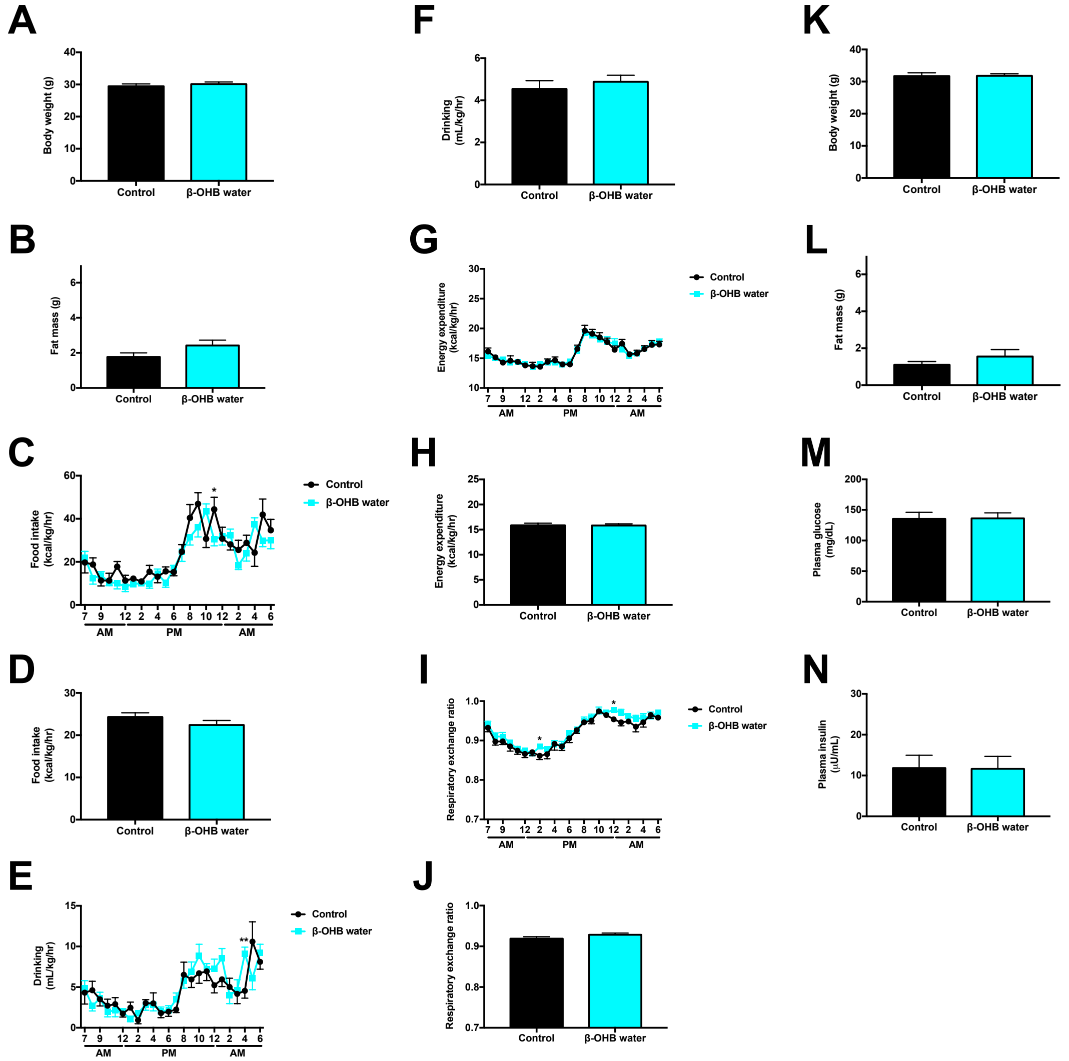

Figure S4. Ketone supplementation in drinking water does not independently alter MC38 colon tumor growth. (A)-(B) Body weight and fat mass one week after tumor implantation. (C) Food intake timecourse. In all panels, **P*<0.05, ***P*<0.01. (D) Daily food intake. (E)-(F) Water drinking timecourse and daily water drinking. (G)-(H) Energy expenditure timecourse and daily energy expenditure. (I)-(J) Respiratory exchange ratio timecourse and daily respiratory exchange ratio. (K)-(L) Body weight and fat mass three weeks after tumor implantation and initiation of β-OHB in drinking water. (M)-(N) Plasma glucose and insulin after three weeks of chronic β-OHB supplementation. All data are the mean±S.E.M. of n=7-8 per group, with groups compared by the 2-tailed unpaired Student's t-test.

Figure S5

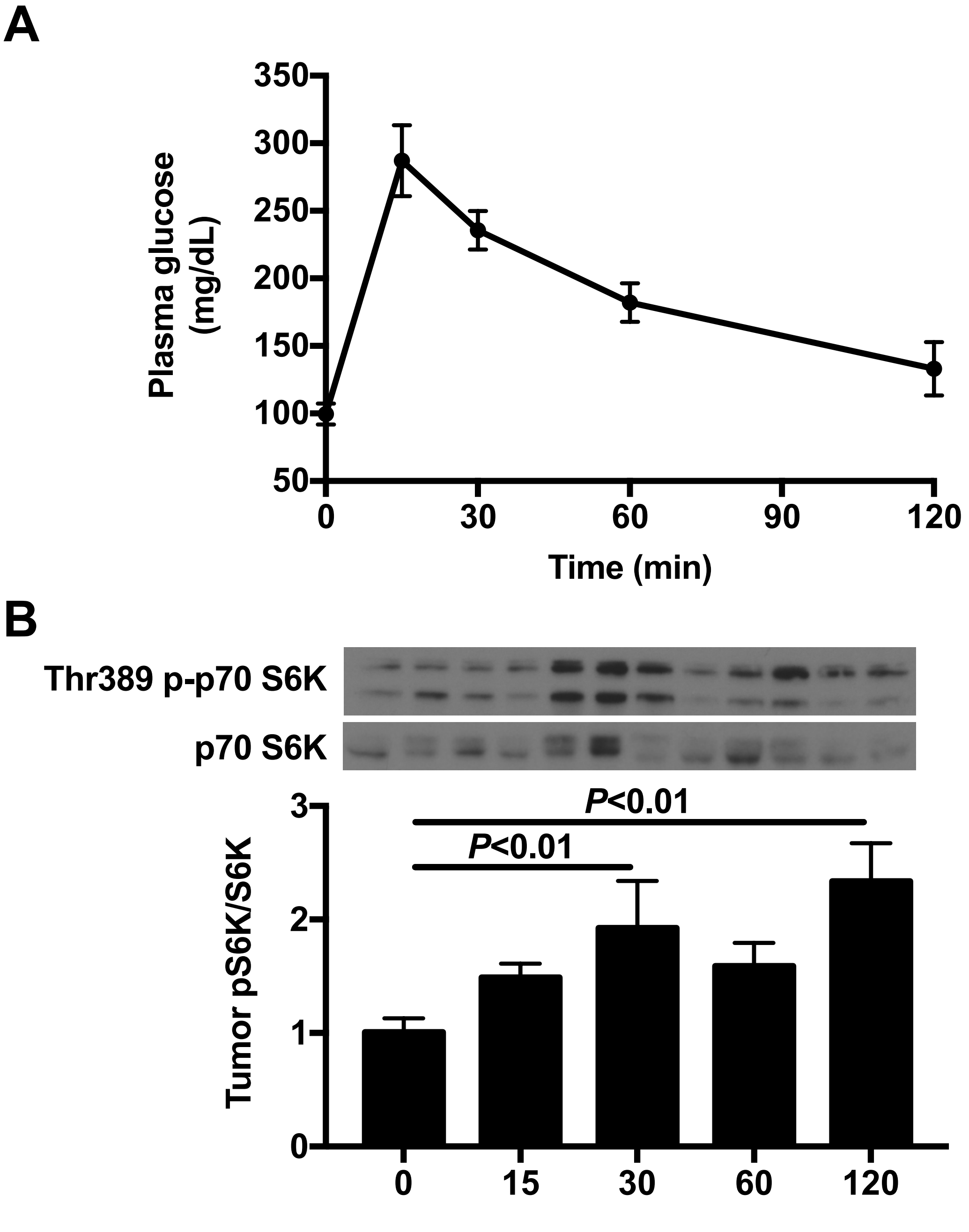

Figure S5. Insulin signaling is dynamically activated under postprandial conditions in MC38 tumors. (A) Plasma glucose. (B) Tumor pS70 S6K pThr389. Both bands of the doublets shown were quantified. Data are the mean ± S.E.M. of n=4-10 per time point, with comparisons by ANOVA with Bonferroni's multiple comparisons test, comparing each group to time zero.
